# Network pharmacology-based investigation of the therapeutic effects and molecular mechanisms of oxymatrine in psoriasis

**DOI:** 10.3389/fphar.2026.1901415

**Published:** 2026-10-26

**Authors:** Yangxu Ding, Wenbo Cai, Haoran Chen, Xiaohan Wang, Yue Ma, Chaowei Liu, Huijuan Shi

**Affiliations:** 1 First Clinical Medical College, Ningxia Medical University, Yinchuan, China; 2 General Hospital of Ningxia Medical University, Yinchuan, China

**Keywords:** apoptosis, EGFR-JAK/STAT signaling pathway, network pharmacology, oxymatrine, psoriasis

## Abstract

**Introduction:**

Psoriasis is a common chronic inflammatory skin disease with complex pathogenesis, and current therapeutic agents have certain limitations. Oxymatrine (OMT), the primary active component of the traditional Chinese herb *Sophora flavescens*, possesses multiple pharmacological effects including anti-inflammation and anti-proliferation, but its molecular mechanism against psoriasis remains incompletely elucidated. This study systematically dissected the “component-target-pathway” regulatory network of OMT against psoriasis using a network pharmacology approach to predict its underlying mechanisms, which were further validated via clinical specimen analysis and *in vitro* cell experiments.

**Methods:**

OMT targets were collected from the Traditional Chinese Medicine Systems Pharmacology (TCMSP) database and literature mining, while psoriasis-related targets were obtained from 5 databases including GeneCards. After target mapping, drug-core target and protein-protein interaction (PPI) networks were constructed, and core targets including EGFR, AKT1, JAK2, and BCL2 were identified. Gene Ontology (GO) and Kyoto Encyclopedia of Genes and Genomes (KEGG) enrichment analyses, molecular docking, and 100 ns molecular dynamics (MD) simulations were conducted. Clinical validation was carried out in patients receiving 8 weeks of OMT treatment. HaCaT keratinocytes were divided into control, M5 cytokine cocktail-induced model, methotrexate (MTX) positive control, and OMT intervention groups for in vitro experiments.

**Results:**

Enrichment analyses revealed significant enrichment in inflammatory response regulation, apoptotic signaling, and multiple JAK/STAT-related pathways. OMT exhibited favorable binding affinities to core targets such as EGFR, JAK1, JAK2, and STAT3. Notably, OMT-JAK1 and OMT-STAT3 complexes demonstrated excellent thermodynamic stability as validated by MD simulations. Clinically, patients’ **Psoriasis Area and Severity Index (PASI)** scores were significantly reduced, lesion histopathology was markedly improved, and aberrant EGFR-JAK/STAT and apoptosis-related molecule expression was significantly reversed. In vitro, OMT significantly inhibited M5-induced EGFR-JAK/STAT phosphorylation and activation, and restored apoptotic balance.

**Discussion:**

These findings suggest that OMT exerts therapeutic effects against psoriasis by inhibiting EGFR phosphorylation, upregulating SOCS1, thereby suppressing excessive JAK/STAT signaling activation, modulating apoptosis-related protein expression, and ultimately promoting keratinocyte apoptosis while inhibiting their proliferation and inflammatory responses. This study provides experimental evidence for the clinical application of OMT in psoriasis treatment.

## 1 Introduction

Psoriasis is a polygenetically determined, immune-mediated hyperproliferative inflammatory skin disorder induced by combined stimulation of multiple environmental factors, clinically characterized by recurrent scaly erythematous plaques covered with multiple layers of dry silvery scales accompanied by varying degrees of pruritus (Griffiths and Barker, 2007). The disease is characterized by frequent relapses and a chronic, relapsing-remitting course, imposing a significant psychological burden on patients, and its exact etiology and pathogenesis remain incompletely understood to date (Scher et al., 2019).

The etiology of psoriasis is complex, and its occurrence and development may involve T cell differentiation (Yin et al., 2026), inflammatory cell infiltration (Zhang et al., 2026), keratinocyte proliferation (Rithvik et al., 2026) and other links. Genetic factors, infections, metabolic disorders, endocrine abnormalities, as well as smoking, alcohol consumption and psychological factors can also induce or even exacerbate the disease (Chiricozzi et al., 2018). Studies have shown that abnormally infiltrated lymphocytes in lesions are one of the key links in the pathogenesis of this disease (Leijten et al., 2021). Cytokines secreted by aberrantly activated T lymphocytes participate in pathological processes such as excessive keratinocyte proliferation and abnormal dermal microvascular proliferation through multiple signaling pathways including JAK/STAT (Wang et al., 2017), NF-κB (Pan et al., 2021) and MAPK (Takahashi et al., 2002). Epidermal Growth Factor Receptor (EGFR), as a key upstream membrane receptor, can be activated by factors such as EGF, TGF-α and IL-17A in the inflammatory microenvironment, which in turn phosphorylates downstream JAK kinases to initiate signaling cascades and forms a cross-regulatory network with NF-κB and MAPK pathways simultaneously. Among them, the JAK/STAT pathway occupies the position of “the central hub for signal convergence and integration”. Like a central processing unit, it receives, integrates and executes instructions from multiple upstream pathways. It can not only directly and rapidly transduce extracellular signals to the nucleus, but also has critical interactions with multiple pathogenic pathways, and its excessive activation is one of the core mechanisms of psoriasis pathogenesis (Cheuk et al., 2014).

Current treatment of psoriasis involves intermittent or long-term use of topical medications. Conventional systemic therapeutic agents mainly include methotrexate, acitretin and cyclosporine, but long-term administration is associated with significant adverse effects and high recurrence rate after discontinuation (Wang et al., 2020; Chong and Fonacier, 2016). Although biological agents have shown remarkable efficacy and rapid onset of action in recent years, they are plagued by problems such as prohibitive cost, requirement for lifelong administration, and high risk of immune tolerance and dysregulation. They may also induce atopic dermatitis and cardiovascular diseases, and are strictly contraindicated in patients with a history of prior infections or malignancies (Armstrong and Read, 2020). Therefore, there is an urgent unmet need to develop novel therapeutic agents with favorable efficacy, high safety profile and minimal side effects.

Oxymatrine (OMT) is a tetracycloquinolizidine alkaloid extracted from the roots of *Sophora alopecuroides* L. (a species of the genus Sophora). It possesses multiple pharmacological activities including anti-inflammatory, anti-fibrotic and immunomodulatory effects, and has been widely used in the treatment of various diseases such as viral hepatitis and myocarditis (Jin et al., 2021; Shi et al., 2010). It has a favorable safety profile with extensive clinical application, and no severe adverse reactions have been reported to date (Kan et al., 2017). Accumulating evidence has demonstrated that OMT can inhibit T lymphocyte activation (Wen and Liu, 2009), alleviate inflammatory injury and regulate cellular apoptosis (Pei et al., 2016; Zhang et al., 2017). Our previous clinical studies have shown that OMT exerts significant therapeutic effects against psoriasis, exhibiting comparable efficacy to acitretin but superior long-term outcomes, with fewer side effects, lower recurrence rate and additional metabolism-regulating functions (Shi et al., 2019; Shi et al., 2016). It can downregulate the expression of PCNA and Ki-67 in skin lesions, modulate the levels of apoptosis-related proteins and promote lesion recovery (Shi et al., 2019). Studies by Xue et al. also suggested that OMT may ameliorate psoriasis symptoms by regulating keratinocyte differentiation (Xue et al., 2022; Xue X. et al., 2023).

The pathogenesis and progression of psoriasis are closely associated with the aberrant activation of the EGFR-JAK/STAT signaling pathway, which serves as the core signaling axis regulating epidermal hyperproliferation and immune dysregulation (Pastore et al., 2014). However, whether OMT can alleviate psoriasis symptoms by modulating this pathway has not been reported to date. Network pharmacology enables the systematic dissection of synergistic interactions among drugs, diseases and targets, and elucidates multi-target and multi-pathway pharmacological mechanisms (Zhai et al., 2025). Therefore, in this study, we employed network pharmacology approaches to predict the potential therapeutic targets of OMT in psoriasis treatment, constructed the “component-target-pathway-disease” network, and validated the findings through clinical specimen analysis and *in vitro* cell experiments, aiming to elucidate its underlying molecular mechanism and provide a theoretical basis for its clinical application.

## 2 Materials and methods

### 2.1 Subject registration, treatment and sampling

A total of 58 patients with severe plaque psoriasis were enrolled between July 2019 and October 2024. All enrolled patients were diagnosed with psoriasis by clinical or histopathological examination. Inclusion criteria: Psoriasis Area and Severity Index (PASI) score ≥12, disease duration ≥6 months, and history of systemic therapy. Exclusion criteria: Patients with special types of psoriasis, severe organ dysfunction, use of immunosuppressants, systemic corticosteroids or retinoids within the past 8 weeks, drug allergy, or pregnant/lactating women. Patients who failed to comply with the medication regimen, discontinued treatment prematurely, or developed severe adverse reactions withdrew from the study. Throughout the 8-week treatment period, all participants were strictly prohibited from using any topical anti-psoriatic agents (including topical glucocorticoids and calcineurin inhibitors) or other systemic therapies for psoriasis. This study was not restricted by gender. All subjects provided written informed consent. The study was conducted in strict accordance with the Declaration of Helsinki and approved by the Ethics Committee of Ningxia Medical University (clinical trial registration number: ChiCTR2100043248).

All 58 patients completed the full course of OMT (trade name: Tianqingfuxin; Zhengda Tianqing Pharmaceutical Co., Ltd., Jiangsu, China) treatment. The drug was prepared as a 0.6 g/100 mL solution and administered intravenously at 100 mL once daily for 8 consecutive weeks. Skin lesion biopsies were obtained from all patients before and after treatment. During the treatment period, changes in skin lesions were dynamically monitored and Psoriasis Area and Severity Index (PASI) scores were calculated. Three patients were randomly selected using the random number table method, and skin lesion samples from the same anatomical site were collected before and after 8 weeks of OMT treatment for validation of relevant study indicators. These samples were used to detect target gene mRNA expression by quantitative real-time polymerase chain reaction (qPCR), as well as target protein expression and localization by Western blotting and immunohistochemistry (IHC). In addition, three normal skin samples were obtained from the Department of Burns and Plastic Surgery to serve as the control group.

### 2.2 Collection and screening of OMT-related target proteins

Putative component targets were retrieved from four databases including Swiss Target Prediction (probability >0), BATMAN, HIT and HERB, followed by removal of duplicate entries.

### 2.3 Collection and screening of psoriasis-related targets

Psoriasis-related genes were retrieved using the keyword “psoriasis” from five databases: GeneCards (Relevance score >10), Comparative Toxicogenomics Database (CTD, Inference Score >10), Pharmacogenomics Knowledgebase (PharmGKB), DrugBank database and Online Mendelian Inheritance in Man (OMIM). Genes collected from the above databases were merged followed by removal of duplicate entries to obtain the final set of psoriasis-related targets. The intersection between OMT targets and psoriasis targets was identified and Venn diagrams were generated using the VennDiagram package in R version 4.3.3 to obtain the potential therapeutic targets of OMT against psoriasis. Subsequently, OMT targets and psoriasis targets were uploaded to the online Venn analysis tool (http://bioinformatics.psb.ugent.be/webtools/Venn/) to confirm the common targets, and corresponding Venn diagrams of overlapping targets were also generated.

### 2.4 Construction of OMT -psoriasis key target network and protein-protein interaction network

First, the intersection target dat (https://cn.string-db.org/); the organism was set to *Homo sapiens*, and the minimum required interaction score was set to 0.4. Network visualization was performed using Cytoscape 3.8.2. The font size, node color intensity, and node size were all set acaset of drugs and diseases was uploaded to the STRING online database (cording to the Degree value. A higher Degree value corresponds to a larger font size, darker node color, and larger node size, indicating a more important role of the node in the protein-protein interaction (PPI) network. Network topological structure analysis was conducted using the CytoNCA plugin.

### 2.5 Gene ontology and kyoto encyclopedia of genes and genomes pathway analysis

Gene Ontology (GO) functional enrichment analysis and Kyoto Encyclopedia of Genes and Genomes (KEGG) pathway enrichment analysis were performed using the online platform Metascape (https://metascape.org/gp/index.html) with the following R packages: org. Hs.eg.db, colorspace, stringi, DOSE, clusterProfiler, pathview, ggplot2 and limma. The significance thresholds were set as pvalueCutoff = 0.05 and qvalueCutoff = 0.05. GO functional analysis included three major categories: Biological Process (BP), Cellular Component (CC) and Molecular Function (MF). The top 10 most significant terms with P < 0.05 in each category were selected for visualization.

### 2.6 Molecular docking validation

Protein sequence information was obtained from the UniProt Knowledgebase (UniProtKB) website (https://www.uniprot.org/uniprotkb), and protein three-dimensional structures were retrieved from the RCSB Protein Data Bank (RCSB PDB) database (https://www.rcsb.org/). The obtained protein structures were imported into Discovery Studio 2019 for structure optimization, which mainly included removal of water molecules, addition of hydrogen atoms, assignment of charges, completion of missing amino acids and completion of missing side chains. Finally, the optimized protein structures were exported as PDB files. The three-dimensional structure of the small molecule (OMT) was downloaded from the PubChem database and subjected to energy minimization using Discovery Studio 2019, then saved as a PDB file. Autodock 4.0 was used to prepare protein and small molecule PDBQT files, and molecular docking was performed using Autodock Vina 1.2.6. Visualization analysis of docking results was conducted using PyMOL 3.1 and Discovery Studio 2019.

### 2.7 Molecular dynamics simulation

Molecular dynamics (MD) simulations of OMT -JAK1 and OMT -STAT3 complexes were performed for 100 ns using GROMACS 2022 software to analyze the binding stability of protein-ligand complexes. The AMBER14SB force field was used for receptor proteins, while the ligand topology files were generated using Sobtop with the GAFF2 force field, and partial charges were assigned using the Restrained Electrostatic Potential (RESP) method. The systems were solvated in a 1 nm cubic water box using the TIP3P water model, and 0.15 M NaCl was added to maintain electrical neutrality and physiological ionic strength. Long-range electrostatic interactions were calculated using the Particle Mesh Ewald (PME) method with a cutoff distance of 1 nm, and bond constraints were applied using the LINCS algorithm. Prior to production simulations, energy minimization was performed sequentially using 3,000 steps of the steepest descent method and 2,000 steps of the conjugate gradient method, followed by full system optimization with staged constraints on solutes and ions. Production simulations were conducted under the NPT ensemble at 310 K (controlled by the Nosé–Hoover thermostat) and 1 bar (controlled by the Parrinello–Rahman barostat) with an integration time step of 2 fs. System stability and structural changes were evaluated by calculating root-mean-square deviation (RMSD), root-mean-square fluctuation (RMSF), hydrogen bonds, radius of gyration (Rg) and solvent-accessible surface area (SASA).

### 2.8 Evaluation of pathological severity of skin lesions by hematoxylin and eosin (HE) staining

Fixed skin tissue samples from skin lesions of each group were paraffin-embedded and sectioned to a thickness of approximately 3 μm. The sections were deparaffinized with xylene according to standard protocols, then rehydrated sequentially in xylene, absolute ethanol, 95% ethanol, 85% ethanol, 75% ethanol, and distilled water, followed by dehydration. Hematoxylin staining was performed for 5 min, followed by differentiation in hydrochloric acid-ethanol for 30 s, bluing in warm water at 50 °C for 5 min, and subsequent dehydration. Eosin staining was conducted for 2 min, followed by dehydration, clearing, and mounting with neutral gum. The sections were observed and photographed under a light microscope.

### 2.9 Immunohistochemical detection of expression and localization of JAKs/STAT3 pathway and related target proteins in skin lesions of patients before and after 8 weeks of treatment

Paraffin sections of skin lesion tissues from patients were deparaffinized with xylene and rehydrated with graded ethanol. Antigen retrieval was performed by boiling in citrate buffer (pH 6.0) for 10 min, followed by incubation with 3% H_2_O_2_ at room temperature for 20 min to block endogenous peroxidase activity. Primary rabbit anti-mouse antibodies were added dropwise at appropriate dilution ratios and incubated at 4 °C for 24 h. Subsequently, biotin-labeled secondary IgG antibodies were added and incubated at room temperature for 2 h. After 3,3′-diaminobenzidine (DAB) development and hematoxylin counterstaining, the sections were mounted with neutral gum. Sections were observed and photographed under a light microscope, and the number of positive cells was counted. The mean optical density (MOD) of positive staining per field, which represents the positive rate, was analyzed using Image-Pro Plus 6.0 software.

### 2.10 Cell culture and psoriasis cell model establishment

HaCaT cells in the logarithmic growth phase were subjected to routine digestion and centrifugation, then resuspended in complete medium. The cell density was adjusted to 1 × 10^5^ cells/mL and seeded into culture plates, followed by incubation in a 37 °C, 5% CO_2_ incubator for 24 h until complete adherence. The old medium was discarded, and cells were gently washed once with PBS. Complete medium containing 2.5 ng/mL M5 cytokine cocktail (IL-1α, IL-17, IL-22, TNF-α, and oncostatin M) was added, with triplicate wells set for each group and a blank control group included. Cells were further cultured for 48–72 h, and cell morphology was observed daily under a microscope. Cells and culture supernatants were collected at the end of induction.

### 2.11 Cytotoxicity assay of OMT and MTX by CCK-8 method

The Cell Counting Kit-8 (CCK-8) assay was used to investigate the effects of OMT and methotrexate (MTX) on HaCaT cell viability. HaCaT cells were seeded into 96-well culture plates at a density of 4 × 10^4^ cells/well and cultured in a CO_2_ incubator until reaching 80% confluence. Subsequently, the medium was replaced with serum-free DMEM medium, and cells were treated with different concentration gradients of OMT (0–3.0 mg/mL) and MTX (0–0.2 μmol/mL), respectively. Eight replicate wells were set for each concentration to ensure the reproducibility and reliability of experimental results. After incubation for 24 h and 48 h, cell viability was detected and evaluated strictly according to the instructions of the CCK-8 kit.

### 2.12 Quantitative real-time polymerase chain reaction (qRT-PCR) for mRNA expression detection

qRT-PCR was performed to detect the mRNA expression levels of related apoptosis proteins in human epidermal tissues and HaCaT cells. For tissue samples, 50 mg of liquid nitrogen-frozen human epidermal tissue was ground into powder in a pre-cooled mortar. For cell samples, the culture medium was discarded, and cells were washed twice with pre-cooled PBS, followed by addition of 1 mL Trizol reagent per well. Total RNA was extracted from both tissues and cells using the standard Trizol method. RNA purity was determined using an ultramicro spectrophotometer, and qualified samples with an A260/A280 ratio of 1.8–2.0 were selected for subsequent experiments. 1 μg of total RNA was reverse transcribed into cDNA, and amplification was performed using the SYBR Green-based method. The reaction program was as follows: pre-denaturation at 95 °C for 30 s, followed by 40 cycles of 95 °C for 5 s and 60 °C for 30 s. Melting curve analysis was performed to verify amplification specificity. GAPDH was used as the internal reference, and the relative gene expression levels were calculated using the 2^−ΔΔCt method. All experiments were independently repeated three times.

### 2.13 Western blotting for protein expression detection

Western blotting was used to detect the expression of JAK/STAT pathway and its upstream and downstream related proteins in human skin lesion tissues and HaCaT cells. For tissue samples, liquid nitrogen-frozen skin lesion tissues were homogenized and lysed on ice in RIPA lysis buffer containing protease and phosphatase inhibitors, followed by centrifugation to collect the supernatant. For cell samples, cells were centrifuged to discard the culture medium, washed with pre-cooled PBS, lysed with RIPA lysis buffer, and centrifuged to obtain the supernatant. Protein concentration was determined using the bicinchoninic acid (BCA) assay, and samples were stored at −80 °C after uniform concentration adjustment. Equal amounts of protein (30 μg) were separated by 10% sodium dodecyl sulfate-polyacrylamide gel electrophoresis (SDS-PAGE) and electroblotted onto polyvinylidene fluoride (PVDF) membranes. The membranes were blocked with 5% non-fat milk at room temperature for 1 h, incubated with primary antibodies at 4 °C overnight, washed with TBST, and then incubated with horseradish peroxidase (HRP)-conjugated secondary antibodies at room temperature for 1 h. After washing again, the membranes were developed with enhanced chemiluminescence (ECL) reagent in the dark for 2 min, and band signals were acquired using an automatic chemiluminescence image analysis system.

### 2.14 Statistical analysis

All data were analyzed using GraphPad Prism 8.0 software and presented as mean ± standard deviation (SD). Student’s t-test was used for comparisons before and after treatment, and p < 0.05 was considered the threshold for statistical significance.

## 3 Results

### 3.1 Prediction of potential therapeutic targets of OMT in psoriasis

In this study, psoriasis-related targets were systematically retrieved from GeneCards, CTD, PharmGkb, OMIM and DrugBank databases. After merging and removing duplicates, a total of 5,616 psoriasis disease targets were obtained. Potential targets of OMT were screened from the TCMSP database, and 241 drug targets were obtained after eliminating invalid and duplicate targets. Standardized conversion of gene IDs and gene symbols for the above targets was performed using the UniProt platform. The intersection of the 241 OMT targets and the 5,616 psoriasis disease targets was calculated, and a Venn diagram was drawn (Figure 1A). A total of 109 common targets of OMT and psoriasis were obtained. Visualization analysis of the above 109 common targets was performed using Cytoscape software, and a drug-key target network diagram was constructed (Figure 1B). The 109 common targets were imported into the STRING database, the organism was set to *H. sapiens*, and a protein-protein interaction (PPI) network was constructed. This network contained 40 nodes and 182 edges (Figure 1C). The top 10 core targets with the highest degree values were screened by topological analysis and a bar chart was drawn (Figure 1D). Among them, AKT1 and EGFR had the highest degree values (both 41), followed by CASP3 (32), MMP9 (31), BCL2 (30), IFNG (27), CYCS (26), PTEN (23), and both PARP1 and CASP1 had a degree value of 21. As a core node, EGFR is an important upstream regulatory molecule of the JAK/STAT pathway. Its signal stimulation can upregulate the phosphorylation of STAT1 at Tyr701 site and induce the formation of complexes through the interaction between STAT1/STAT3 and JAK1/JAK2 (Andl et al., 2004). In addition, AKT1 enhances pathway signal transduction through phosphorylation (Lu et al., 2008); CASP3 and STAT3 cross-regulate the balance of cell apoptosis (Li et al., 2024); MMP9 degrades the extracellular matrix to promote inflammatory infiltration and indirectly activates the JAK/STAT pathway (Nishikai-Yan Shen et al., 2021); BCL2 is regulated by STAT3, which inhibits cell apoptosis and maintains abnormal proliferation of keratinocytes (Wang et al., 2024); IFNG activates the JAK1-STAT1/STAT3 pathway to aggravate inflammation (Han et al., 2023). Taken together, the core target proteins regulate the JAK/STAT pathway in a multi-dimensional manner with EGFR as the core. OMT may exert therapeutic effects by targeting these hub targets, reversing the imbalance of keratinocyte proliferation and inflammation in psoriasis, suggesting that OMT may block the excessive activation of the JAK/STAT pathway by targeting EGFR.

**FIGURE 1:**
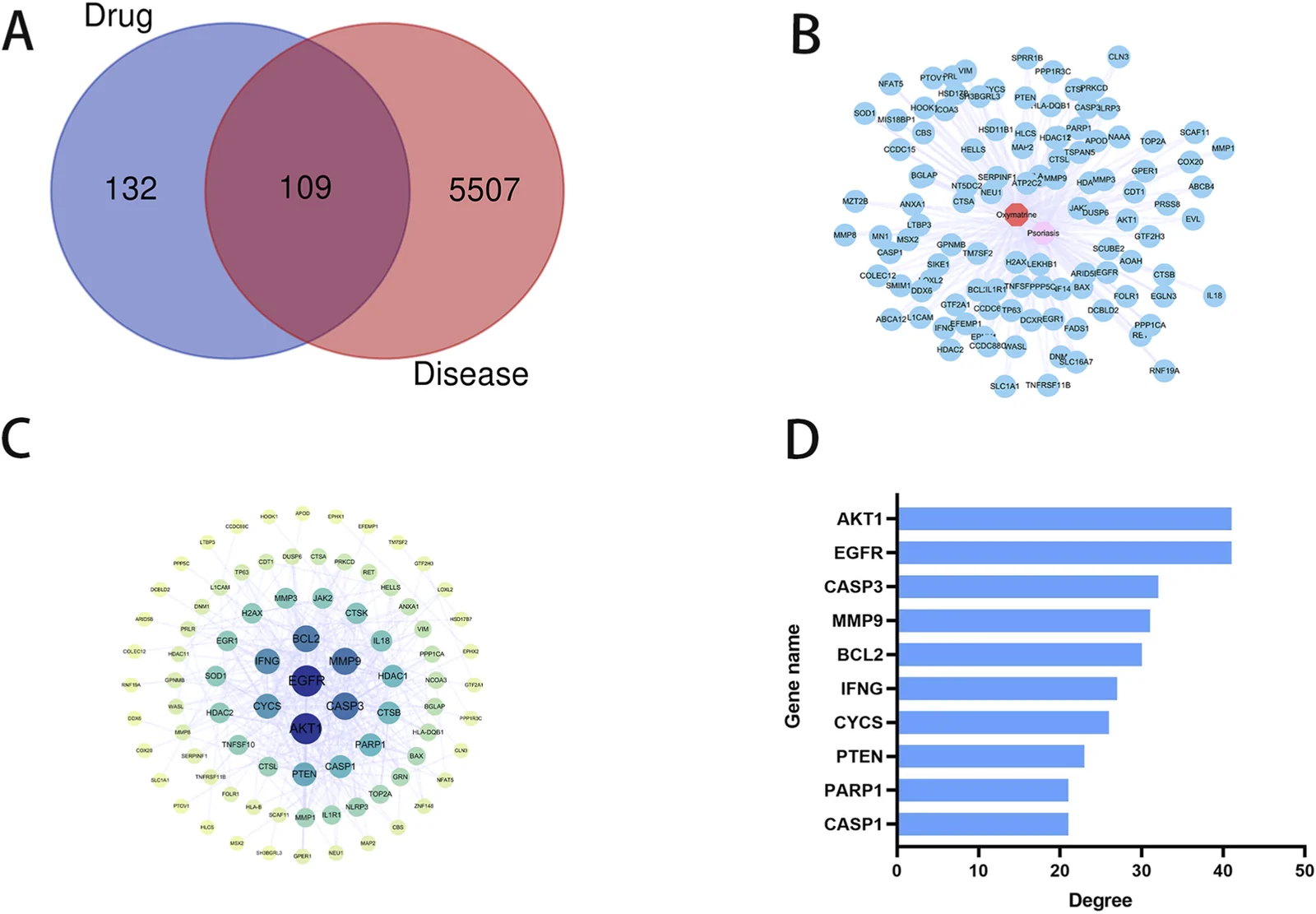
Network pharmacology analysis of potential targets and molecular mechanisms of OMT in the treatment of psoriasis. **(A)** Venn diagram of OMT-related targets and psoriasis-related targets. The blue circle represents 241 predicted targets of OMT, and the red circle represents 5,616 psoriasis disease targets. The overlapping region contains 109 common therapeutic targets. **(B)** Protein-protein interaction (PPI) network of the 109 intersecting targets. Nodes represent proteins, and edges represent interactions between proteins. Node size and color intensity are positively correlated with the degree value of the target. **(C)** PPI network of core hub genes screened from the intersecting targets. The top 10 hub genes ranked by degree value are highlighted with larger nodes and darker colors. **(D)** Bar chart of the degree values of the top 10 hub genes. The horizontal axis represents the degree value, and the vertical axis represents the gene name.

### 3.2 GO and KEGG enrichment analysis

Gene Ontology (GO) functional annotation was performed on the common targets of OMT and psoriasis, and enrichment analysis was conducted from three aspects: Biological Process (BP), Cellular Component (CC) and Molecular Function (MF). The results were presented as bubble plots (Figures 2A–C). In the BP category (Figure 2A), terms such as regulation of inflammatory response, cellular response to oxidative stress and regulation of apoptotic signaling pathway were the most significantly enriched, which were highly consistent with the core pathological features of psoriasis including persistent inflammatory infiltration, oxidation-antioxidation imbalance and abnormal keratinocyte apoptosis. This suggests that OMT exerts therapeutic effects by synergistically intervening in these processes through multiple targets. The enrichment of terms related to regulation of neuronal death may be associated with abnormal neuropeptide release and neurogenic inflammation in skin lesions. In the CC category (Figure 2B), components of the endosome-lysosome system such as endosomal lumen and endolysosome were the most significantly enriched, indicating that key targets are concentrated in intracellular membrane trafficking and degradation systems. These are closely related to antigen presentation in psoriasis (Harden et al., 2015), pattern recognition receptor signal transduction (Sun et al., 2019) and autophagy regulation (Yin et al., 2018), and participate in intracellular immune-inflammatory signal regulation. The term of collagen-containing extracellular matrix corresponds to skin lesion tissue remodeling (Xu et al., 2026). In the MF category (Figure 2C), histone deacetylase-related activities were the most significantly enriched, suggesting that OMT may affect the transcription of psoriasis-related genes by regulating histone acetylation modifications. Abnormalities in this epigenetic regulatory link have been confirmed to be involved in the pathogenesis of psoriasis (Liang et al., 2025). The enrichment of serine-type peptidase/hydrolase activities (Czerwinska and Owczarczyk-Saczonek, 2025) is related to the proteolytic activation of cytokine precursors and tissue remodeling processes during inflammation. Taken together, the functions of key targets of OMT are highly concentrated in core pathological processes such as inflammation regulation, oxidative stress response, apoptosis regulation and epigenetic modification, providing a functional annotation basis for its multi-target therapeutic effects.

**FIGURE 2:**
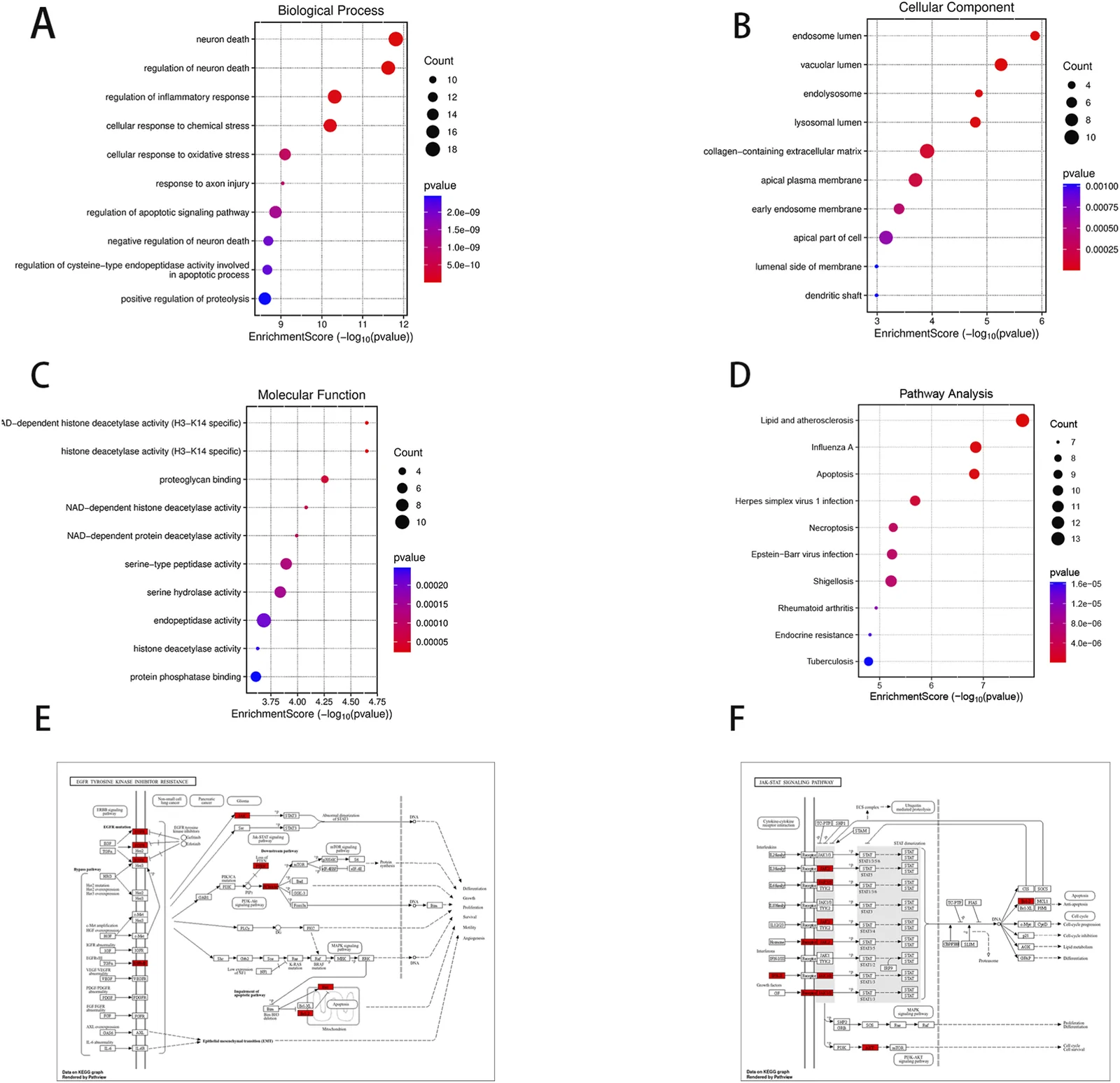
Functional enrichment and core pathway mechanism analysis of intersecting targets of OMT in the treatment of psoriasis. **(A–C)** Gene Ontology (GO) functional enrichment analysis of intersecting targets, including **(A)** Biological Process (BP), **(B)** Cellular Component (CC), and **(C)** Molecular Function (MF). The horizontal axis represents the enrichment score [−log10 (P value)], and the vertical axis represents the enriched functional terms. The size of the dots represents the number of enriched genes, and the color gradient of the dots represents the significance level of the P value (from blue to red indicates gradually increasing significance). **(D)** Kyoto Encyclopedia of Genes and Genomes (KEGG) signaling pathway enrichment analysis of intersecting targets. The horizontal axis represents the enrichment score [−log10 (P value)], and the vertical axis represents the enriched signaling pathways. The size of the dots represents the number of enriched genes, and the color gradient of the dots represents the significance level of the P value (from blue to red indicates gradually increasing significance). **(E)** Mechanism diagram of the EGFR pathway. **(F)** Mechanism diagram of the JAK-STAT pathway.

KEGG pathway enrichment analysis was performed using the KEGG database. A total of 221 functional pathways were obtained, of which 99 were disease-related. Ranked by P-value, the top 10 enriched pathways included apoptosis, necroptosis, rheumatoid arthritis, lipid and atherosclerosis, and multiple infection-related pathways (Figure 2D). All the above pathways were closely related to the pathogenesis of psoriasis and had extensive cross-regulation with the JAK/STAT signaling pathway: Apoptosis and necroptosis directly targeted the core imbalance of keratinocyte proliferation-apoptosis (Qu et al., 2023; Chen et al., 2025); the rheumatoid arthritis pathway shared the Th17/IL-23 immune-inflammatory network with psoriasis (Schinocca et al., 2021); the lipid and atherosclerosis pathway reflected the metabolic inflammatory state associated with psoriasis (Ryu et al., 2019; Zhen et al., 2024; Shen et al., 2023); infection-related pathways indicated that targets were distributed at key nodes of the host immune response (Low et al., 2022; Sato et al., 2026). All pathways could converge on the JAK/STAT signaling axis at key nodes, forming a cross-regulatory network centered on this pathway. This finding was mutually corroborated with the results that JAK2 and EGFR were core nodes in the PPI network, suggesting that OMT may synergistically intervene in the JAK/STAT signaling axis through EGFR and multiple pathways, regulate the imbalance of inflammation and cell proliferation-apoptosis in psoriasis, and provide bioinformatics evidence for subsequent mechanism verification. Mechanism diagrams of the EGFR and JAK-STAT pathways (Figures 2E,F).

### 3.3 Molecular docking and molecular dynamics simulation

To investigate the binding characteristics between OMT and the core targets of psoriasis, molecular docking technology was used to perform ligand-receptor docking analysis between OMT and eight key targets including EGFR, JAK1, JAK2 and STAT3 (Figures 3A–H). The lower the binding free energy, the stronger the binding affinity between the ligand and the target protein, and the more stable the conformation of the complex. The results showed (Table 1; Figure 2A) that OMT had the lowest binding energy of −8.3 kcal/mol with EGFR, showing the optimal binding activity; the binding energy with JAK2 was −8.0 kcal/mol. Both of these values were below −8.0 kcal/mol, meeting the standard of extremely strong binding activity. The binding energies with BCL2 (−7.9 kcal/mol), JAK1 (−7.7 kcal/mol), STAT3 (−7.1 kcal/mol) and SOCS1 (−7.1 kcal/mol) were in the range of −7.0 to −8.0 kcal/mol, meeting the standard of effective and stable binding. The binding stabilities with BAX (−6.9 kcal/mol) and CASP3 (−6.1 kcal/mol) were relatively limited. Focusing on the EGFR-JAK/STAT signaling axis, the core nodes at its upstream, juxtamembrane and downstream regions showed a gradient binding characteristic, suggesting that OMT can exert a synergistic intervention effect by simultaneously occupying key targets at multiple levels of the pathway. The good binding activity of apoptosis regulatory proteins provides a molecular basis for the execution of downstream effects of the pathway. Taken together, molecular docking preliminarily verified that OMT can widely target core nodes at multiple levels of the EGFR-JAK/STAT axis and apoptosis regulatory proteins, providing structural chemical support for its mechanistic hypothesis of regulating the imbalance of keratinocyte proliferation-apoptosis in psoriasis.

**FIGURE 3:**
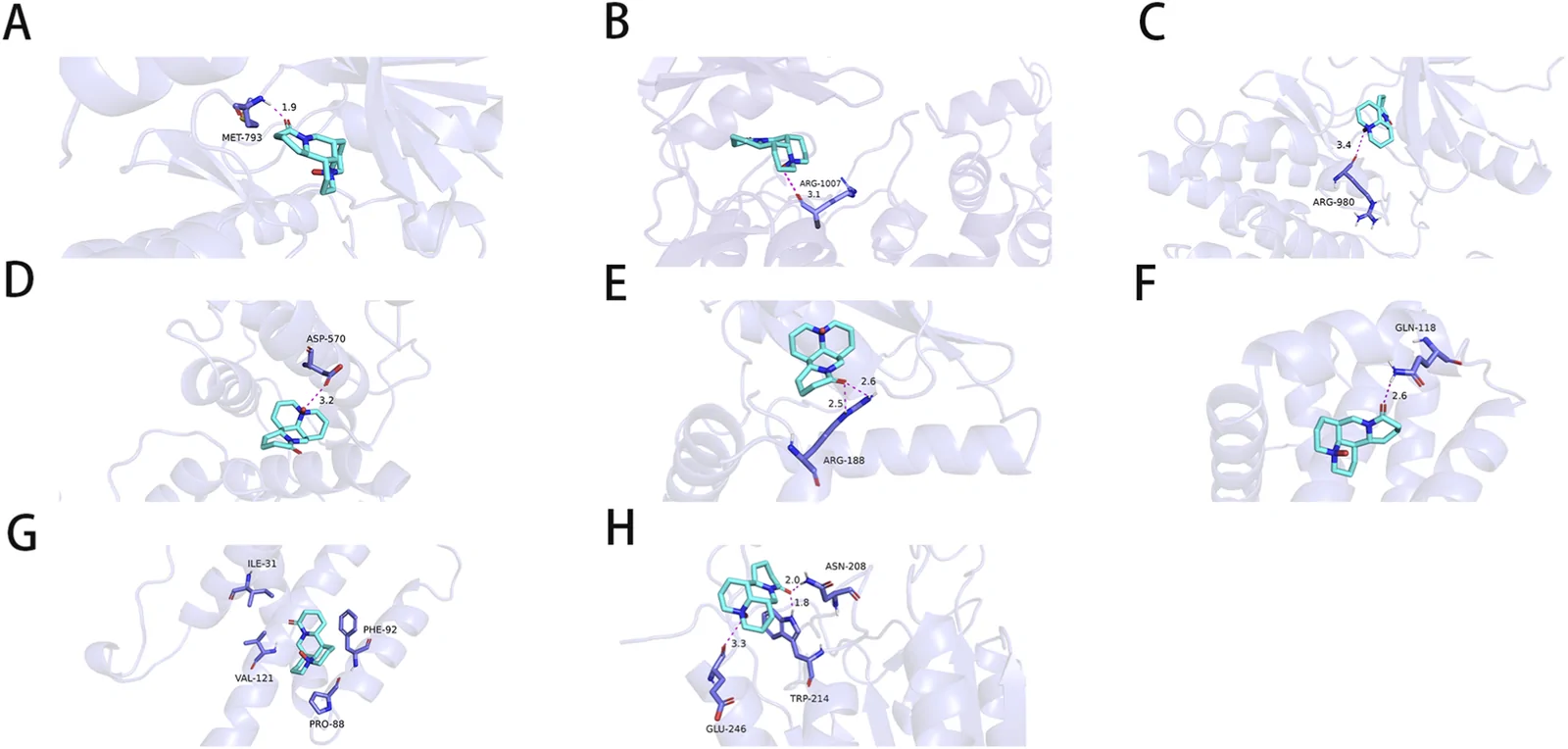
Molecular docking binding mode analysis of OMT with core hub targets of psoriasis. **(A–H)** Molecular docking binding mode diagrams of OMT with EGFR, JAK1, JAK2, STAT3, SOCS1, BCL2, BAX and CASP3, respectively. The stick structure represents the OMT molecule, the ring structure represents the key amino acid residues involved in the interaction in the target protein, and the dashed line represents the hydrogen bond interaction. The value marked next to the dashed line is the hydrogen bond length (unit: Å). The results showed that OMT could form stable hydrogen bond binding with the active pockets of the above 8 core targets, further verifying the reliability of the network pharmacology screening results.

**TABLE 1:** Summary of docking binding energies and PDB IDs for key targets.

Treatment targets	PDB id	Binding energies (kcal/mol)
EGFR	1XKK	−8.3
JAK1	6N7A	−7.7
JAK2	2B7A	−8.0
STAT3	6NJS	−7.1
SOCS1	6C7Y	−7.1
BCL2	6GL8	−7.9
BAX	4BD2	−6.9
CASP3	1QX3	−6.1

**TABLE 2:** Molecular docking grid parameters.

Target protein	Grid center X (Å)	Grid center Y (Å)	Grid center Z (Å)	Grid size X (Å)	Grid size Y (Å)	Grid size Z (Å)
EGFR	17.60	32.60	40.17	31.64	31.64	31.64
JAK1	6.40	−22.82	12.20	47.25	42.00	47.25
JAK2	117.79	65.21	5.84	25.00	25.00	25.00
STAT3	17.25	21.70	20.40	41.25	41.25	41.25
SOCS1	−3.60	2.27	−0.84	46.38	46.38	46.38
BCL2	3.43	0.30	9.03	41.25	41.25	41.25
BAX	25.93	−1.07	46.00	36.55	36.55	36.55
CASP3	6.65	36.64	2.64	42.48	42.48	42.48

To verify the dynamic stability of the binding between OMT and the core targets of the JAK/STAT pathway, JAK1 and STAT3 with strong binding affinity were selected for molecular dynamics simulation. The conformational stability of the complexes was evaluated by root-mean-square deviation (RMSD), radius of gyration (Rg), solvent-accessible surface area (SASA), hydrogen bonds, and root-mean-square fluctuation (RMSF). The results showed that the RMSD of the JAK1-OMT complex reached equilibrium after 15 ns and stabilized at approximately 1.8 Å (Figure 4A); the RMSD of the STAT3-OMT complex fluctuated smoothly throughout the simulation and was always below 2.8 Å (Figure 4F). Both complexes exhibited excellent conformational stability. Rg and SASA analyses indicated that the protein structures remained compact during the simulation, with no significant changes before and after binding (Figures 4B,C,I,J). Hydrogen bond analysis revealed that the number of hydrogen bonds in the JAK1-OMT complex was predominantly 1 (fluctuating between 0 and 4), while that in the STAT3-OMT complex was predominantly 1–2 (fluctuating between 0 and 3). The continuous dynamic hydrogen bond interactions played a crucial role in the stable binding of the ligand (Figures 4D,I). RMSF analysis demonstrated that the RMSF values of amino acid residues in both complexes were mostly below 4 Å, indicating stable local conformations (Figures 4E,J). Taken together, OMT can form stable complexes with JAK1 and STAT3 in dynamic aqueous solution, confirming its ability to target multiple hierarchical nodes of the JAK/STAT pathway at the dynamic molecular level. Combined with the extremely strong binding activity of EGFR observed in the previous molecular docking, this further supports that OMT possesses the structural basis for multi-target synergistic intervention along the EGFR-JAK-STAT signaling axis.

**FIGURE 4:**
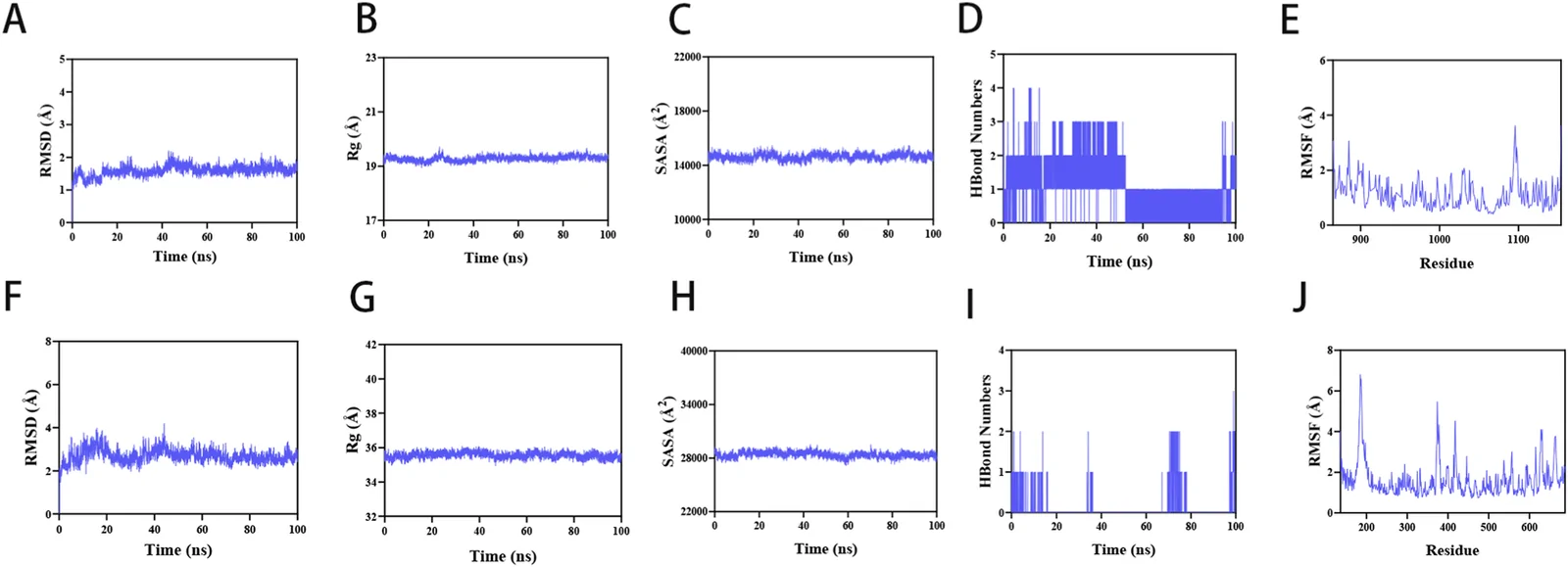
Stability analysis of 100 ns molecular dynamics simulations of OMT-JAK1 and OMT-STAT3 complexes. **(A–D)** Kinetic stability parameters of the OMT-JAK1 complex: **(A)** Root Mean Square Deviation (RMSD) of the main chain; **(B)** Radius of Gyration (Rg); **(C)** Solvent Accessible Surface Area (SASA); **(D)** Curve of the number of intermolecular hydrogen bonds over simulation time. **(E)** Root Mean Square Fluctuation (RMSF) analysis of JAK1 protein residues in the OMT-JAK1 complex. **(F–J)** Kinetic stability parameters of the OMT-STAT3 complex: **(F)** Main chain RMSD; **(G)** Rg; **(H)** SASA; **(I)** Curve of the number of intermolecular hydrogen bonds over simulation time; **(J)** RMSF analysis of STAT3 protein residues. The results showed that both complexes rapidly reached equilibrium and remained stable during the 100 ns simulation, with small fluctuations in RMSD, Rg and SASA values, and could form stable intermolecular hydrogen bonds, confirming that OMT has good binding stability with JAK1 and STAT3 targets.

### 3.4 PASI score and hematoxylin and eosin (HE) staining

A total of 58 patients with severe plaque psoriasis were enrolled between July 2019 and October 2024 and treated with OMT infusion (0.6 g/100 mL) for 8 weeks, followed by a 6-month follow-up observation. After treatment with OMT, the PASI score of the 58 psoriasis patients significantly decreased from 29.0 ± 7.4 at baseline to 7.6 ± 4.7 (P < 0.0001), indicating that both the area and severity of skin lesions were significantly improved after OMT intervention (Figures 5A,B). HE staining was performed on skin lesion tissues before and after treatment. Before treatment, the skin lesions of patients showed typical pathological changes of psoriasis: epidermal hyperkeratosis with focal parakeratosis, thinning or disappearance of the granular layer, significant acanthosis, and regular downward extension of epidermal rete ridges in a club-like or rod-like shape; the dermal papillary layer was widened and edematous, with tortuous and dilated capillaries, and infiltration of inflammatory cells such as lymphocytes and neutrophils. After OMT treatment, the above pathological changes were significantly alleviated, with improved epidermal hyperkeratosis and acanthosis, reduced inflammatory cell infiltration, and the epidermal and dermal structures tended to be normal (Figures 5C,D). Three typical patients were randomly selected using the random number table method. Their PASI scores decreased from 26.4 ± 8.6 before treatment to 10.2 ± 3.7 after treatment (P = 0.0103). Intergroup comparisons showed that there were no statistically significant differences in baseline characteristics before treatment and therapeutic efficacy after treatment between the three patients and the overall patient population (all P > 0.05), indicating that the skin lesions of the three patients could represent the overall population for subsequent validation experiments.

**FIGURE 5:**
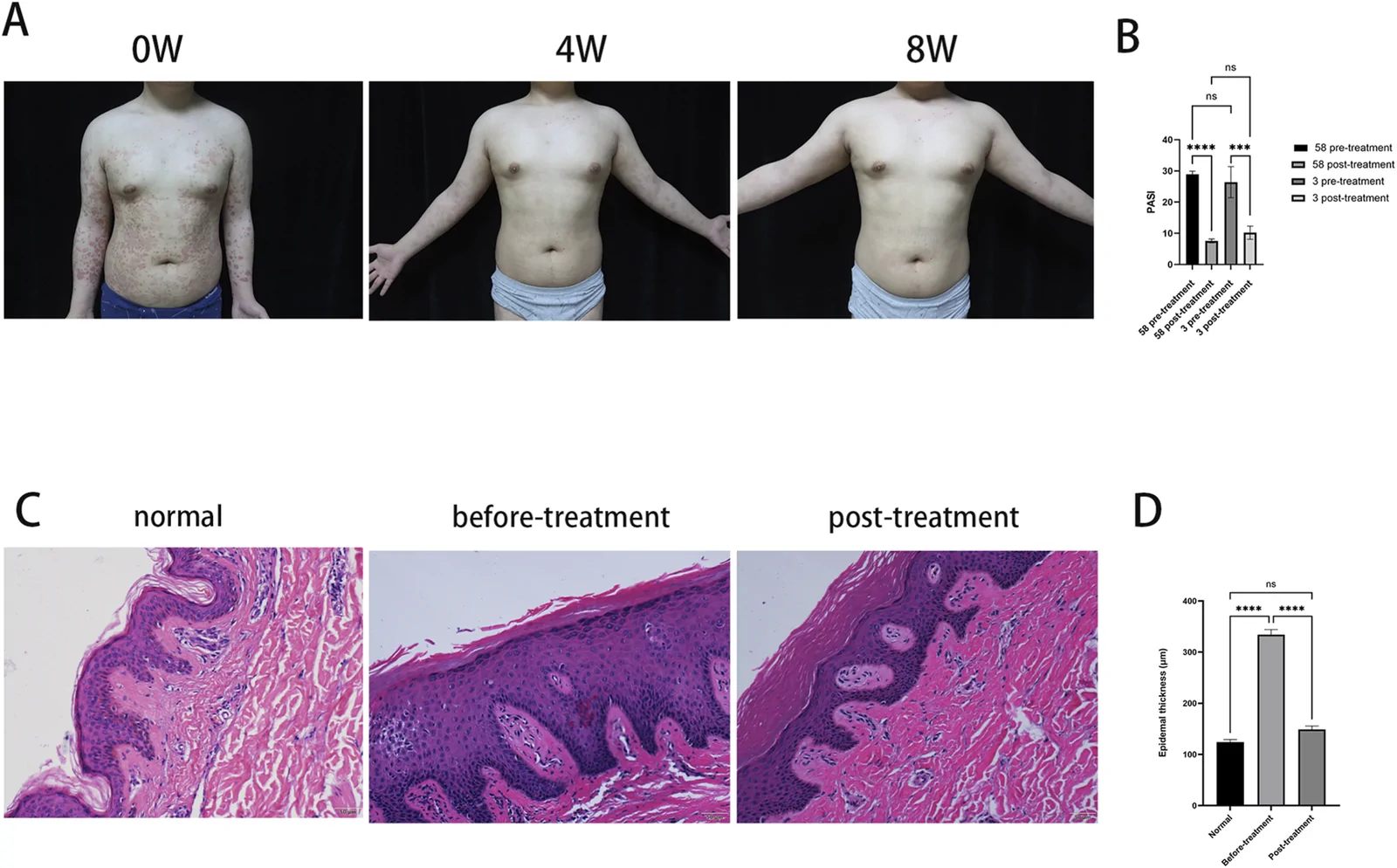
Clinical efficacy and histopathological improvement of OMT in the treatment of psoriasis. **(A)** Representative photographs of trunk skin lesions in a psoriasis patient before and after 0, 2, 4, 6, and 8 weeks of OMT treatment, showing progressive regression of erythema, scales, and infiltration of skin lesions with prolonged treatment time. **(B)** Psoriasis Area and Severity Index (PASI) scores of 58 enrolled psoriasis patients and three patients used for histopathological examination before and after treatment. ns: no statistically significant difference; ****P < 0.0001. **(C)** Hematoxylin-Eosin (HE) staining results of skin tissues (×20), showing normal control skin, pre-treatment skin lesions of psoriasis patients, and skin lesions after 8 weeks of OMT treatment from left to right. **(D)** Quantitative analysis of epidermal thickness in normal control skin, pre-treatment skin lesions, and post-treatment skin lesions (unit: μm). ns: no statistically significant difference; n = 3, ****P < 0.0001. The results showed that OMT treatment could significantly improve the clinical skin lesion symptoms of psoriasis patients, reduce PASI scores, inhibit excessive epidermal hyperplasia, and promote the restoration of normal histopathological structure of skin tissues.

### 3.5 Immunohistochemistry

The results showed (Figure 6) that compared with baseline, the positive expression rates of JAKs/STAT3 pathway-related proteins phosphorylated JAK1 (p-JAK1), phosphorylated JAK2 (p-JAK2) and phosphorylated STAT3 (p-STAT3), their upstream molecule EGFR, and apoptosis-related protein Bcl-2 were significantly decreased in the skin lesions of patients after 8 weeks of OMT treatment, while the positive expression rates of SOCS1, Bax and CASP3 proteins were significantly increased, with all differences being statistically significant (P < 0.05).

**FIGURE 6:**
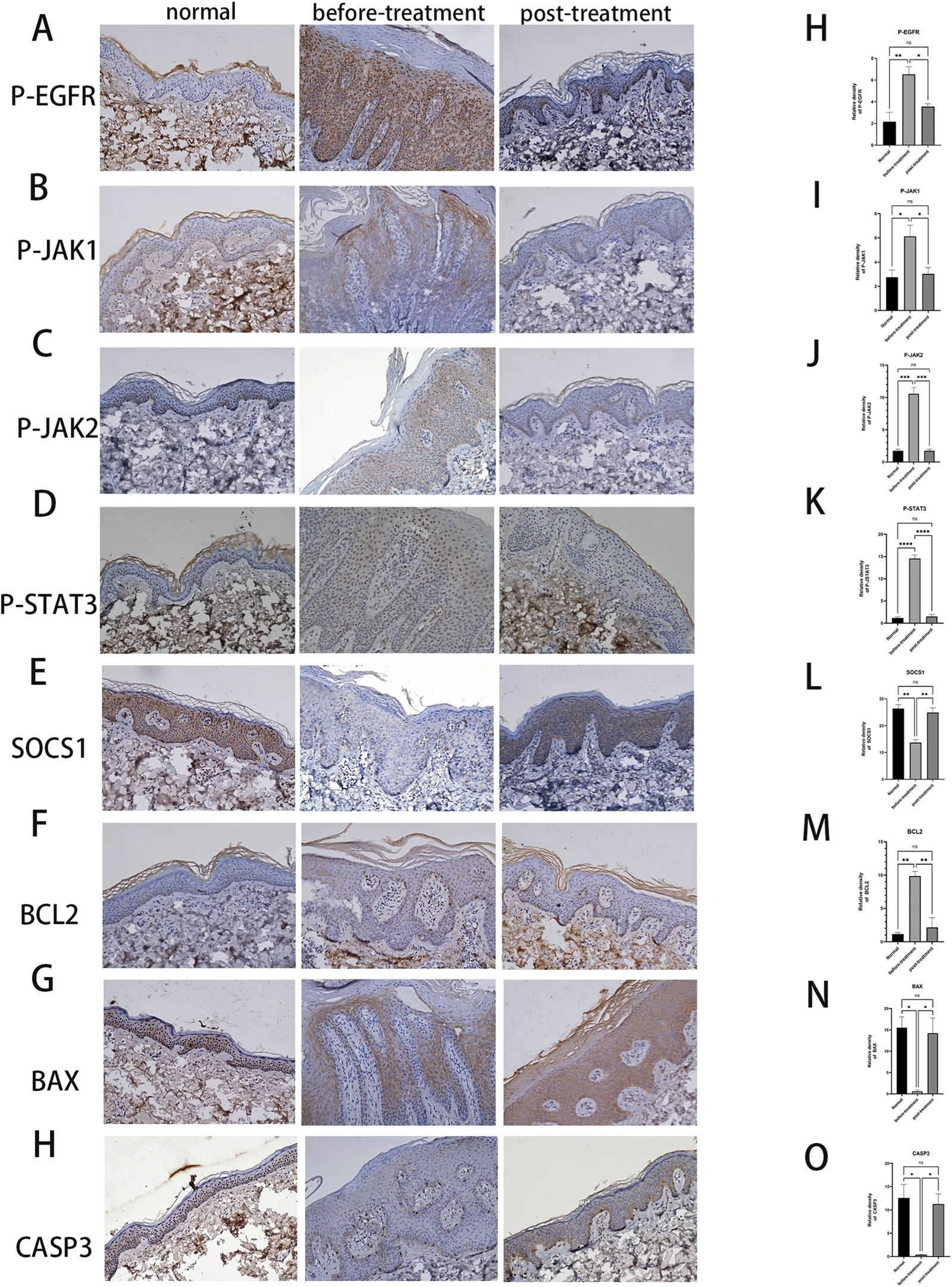
Expression of EGFR-JAK/STAT pathway and apoptosis-related proteins in skin lesion tissues of psoriasis patients after OMT intervention detected by immunohistochemistry. **(A–H)** Immunohistochemical staining was used to detect the expression levels of P-EGFR, P-JAK1, P-JAK2, P-STAT3, SOCS1, BCL2, BAX and CASP3 proteins in normal skin, pre-treatment skin lesions of psoriasis patients and skin lesions after 8 weeks of OMT treatment (×20). Brownish-yellow staining represents positive expression. **(I–O)** Semi-quantitative analysis results of the above protein expressions, expressed as mean optical density (MOD) values. n = 3, *P < 0.05, **P < 0.01, ***P < 0.001, ****P < 0.0001; ns: no statistically significant difference.

### 3.6 Western blotting and qRT-PCR detection of JAKs/STAT3 pathway and its upstream and downstream protein expression in skin lesion tissues

qRT-PCR was performed to detect the mRNA expression levels of EGFR-JAK/STAT pathway-related molecules in psoriasis skin lesion tissues before and after treatment (Figure 7A). The results showed that the mRNA expression of SOCS1, a negative feedback regulator of the pathway, was significantly upregulated after treatment (P < 0.05), consistent with the trend in the normal control group; among the downstream apoptosis regulatory molecules, the mRNA expressions of pro-apoptotic factors CASP3 and Bax were significantly increased (P < 0.05), while the mRNA expression of anti-apoptotic factor Bcl-2 was significantly decreased (P < 0.05), all approaching the levels in the normal control group. Western blotting was used to detect the expression of pathway and apoptosis-related proteins in skin lesion tissues before and after treatment (Figures 7B,C). After 8 weeks of OMT treatment, the protein expressions of p-JAK1, p-JAK2, p-STAT3 and upstream EGFR in the skin lesions of patients were significantly decreased compared with those before treatment (P < 0.05), while the protein expression of SOCS1 was significantly increased (P < 0.05); among the apoptosis-related proteins, the expression of Bcl-2 was significantly decreased, while the expressions of Bax and CASP3 were significantly increased (P < 0.05). These results indicated that OMT could effectively inhibit the overactivated EGFR-JAK/STAT signaling axis in psoriasis skin lesions at the protein and phosphorylation activity levels, and simultaneously upregulate SOCS1 to enhance negative regulation. Concomitant with the inhibition of pathway activity, the downstream pro-apoptotic/anti-apoptotic balance shifted towards the pro-apoptotic direction, consistent with the trend of qRT-PCR results, collectively supporting the molecular mechanism that OMT restores the apoptotic homeostasis of keratinocytes by inhibiting this pathway at both the protein and post-translational modification levels.

**FIGURE 7:**
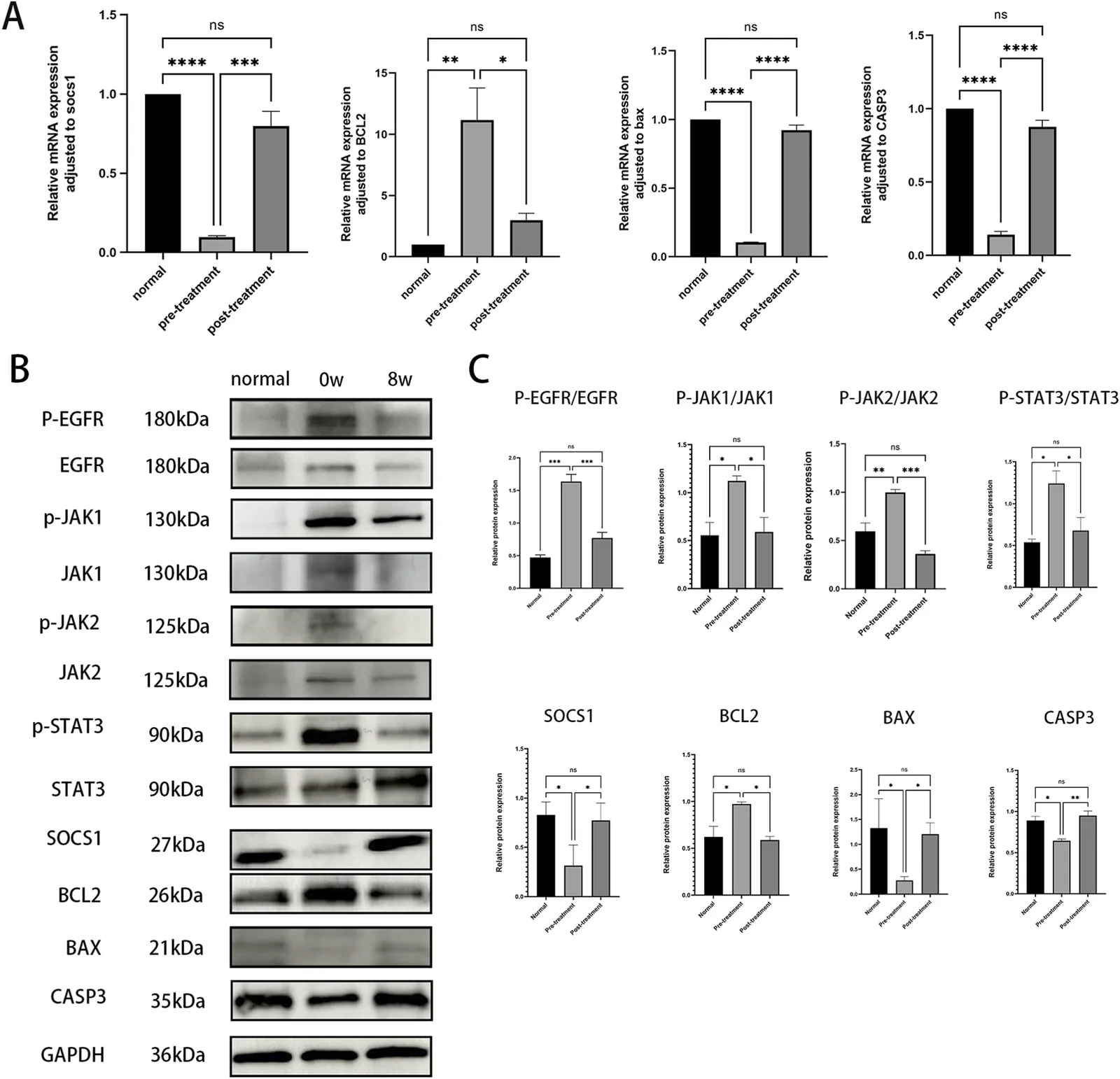
Regulatory effects of OMT on the transcriptional and translational levels of EGFR-JAK/STAT pathway and apoptosis-related molecules in skin lesion tissues of psoriasis patients. **(A)** Quantitative Real-Time Polymerase Chain Reaction (qRT-PCR) was used to detect the mRNA expression levels of SOCS1, BCL2, BAX and CASP3 in normal skin, pre-treatment skin lesions of psoriasis patients and skin lesions after 8 weeks of OMT treatment. **(B)** Western blotting was used to detect the protein expression levels of p-EGFR/EGFR, p-JAK1/JAK1, p-JAK2/JAK2, p-STAT3/STAT3, SOCS1, BCL2, BAX and CASP3 in the above three groups of skin lesion tissues. GAPDH was used as the internal reference protein, and the molecular weights of each protein (unit: kDa) are labeled on the left. **(C)** Quantitative analysis results of gray values corresponding to the above protein expressions. n = 3, *P < 0.05, **P < 0.01, ***P < 0.001, ****P < 0.0001; ns: no statistically significant difference.

### 3.7 CCK-8 assay results

Taking the 24 h time point as an example (Figure 8A), the cytotoxicity of OMT treatment at concentrations ranging from 0.5 to 3.0 mg/mL was evaluated in HaCaT keratinocytes. As shown in the figure, OMT treatment resulted in a gradual decrease in cell viability in a dose-dependent manner, and the viability remained relatively high (≥88.0%) at concentrations up to 1.5 mg/mL, with no statistically significant difference compared with untreated HaCaT cells (P > 0.05). However, at 2.0 mg/mL, the viability decreased to 69.2%, with a statistically significant difference (P < 0.05). A more pronounced decrease was observed at 3.0 mg/mL, with viability dropping to 55.2%. To minimize cytotoxicity while ensuring reliable experimental conditions, concentrations up to 1.5 mg/mL were used in subsequent experiments. Similarly, taking the 24 h time point as an example (Figure 8C), the cytotoxicity of MTX treatment at concentrations ranging from 0.02 to 0.2 μmol/mL was evaluated in HaCaT keratinocytes. As shown in the figure, MTX treatment resulted in a gradual decrease in cell viability in a dose-dependent manner, and the viability remained relatively high (≥93.1%) at concentrations up to 0.1 μmol/mL, with no statistically significant difference compared with untreated HaCaT cells (P > 0.05). However, at 0.12 μmol/mL, the viability decreased to 69.0%, with a statistically significant difference (P < 0.05). A more pronounced decrease was observed at 0.2 μmol/mL, with viability dropping to 55.3%. To minimize cytotoxicity while ensuring reliable experimental conditions, concentrations up to 0.1 μmol/mL were used in subsequent experiments.

**FIGURE 8:**
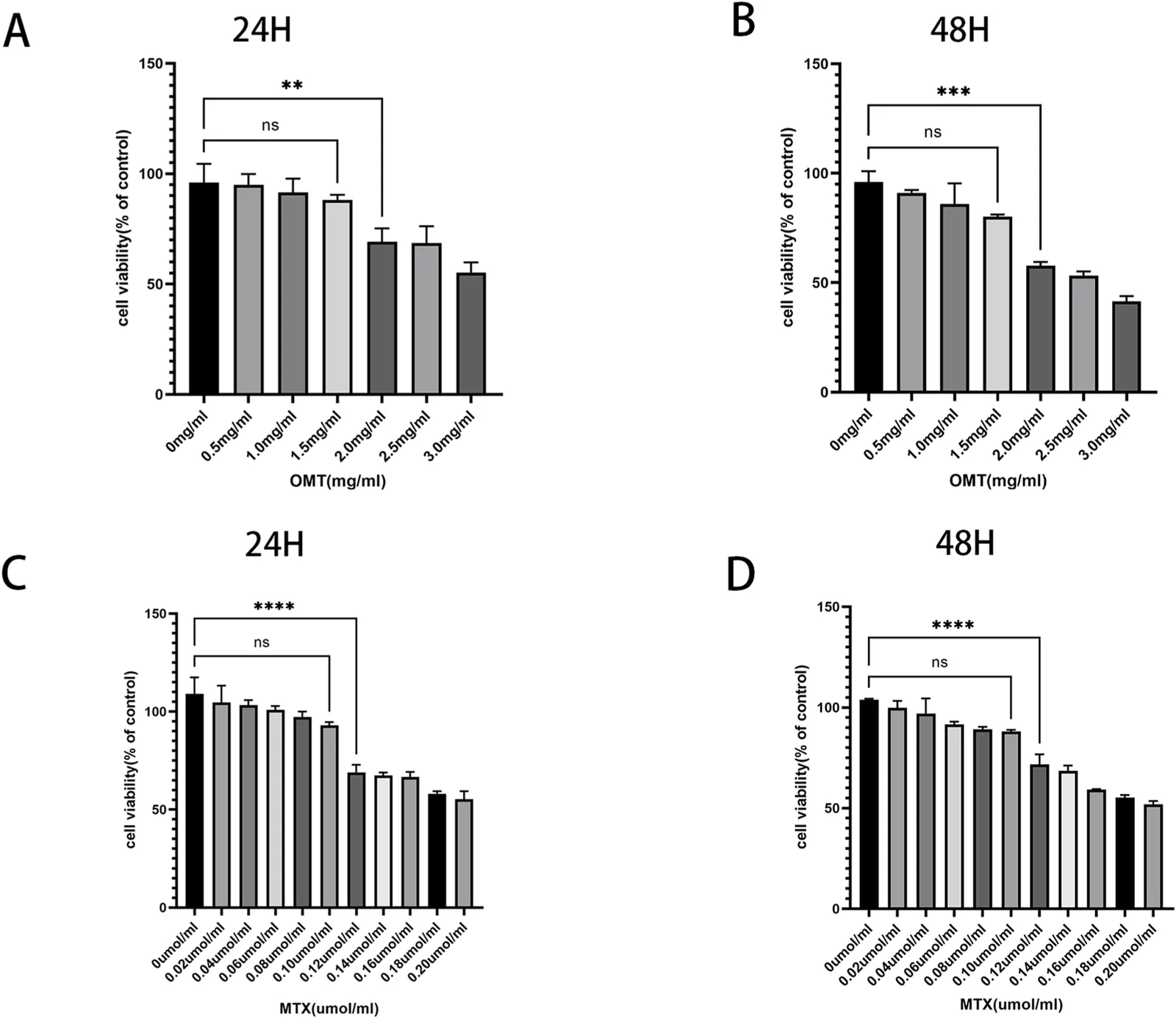
Cell viability detected by CCK-8 assay after treatment with different concentrations of OMT or methotrexate (first-line positive control for psoriasis) for 24 h and 48 h. **(A, B)** Cell viability after OMT treatment for 24 h **(A)** and 48 h **(B)**. **(C, D)** Cell viability after MTX treatment for 24 h **(C)** and 48 h **(D)**. Cell viability data were collected from three independent biological replicates (n = 3).

### 3.8 Western blotting and qRT-PCR detection of JAKs/STAT3 pathway and its upstream and downstream protein expression in HaCaT cells

qRT-PCR was performed to detect the mRNA expression of pathway-related molecules in different treatment groups (Figure 9A). The results showed that the mRNA levels of EGFR and core molecules of the JAK/STAT pathway in HaCaT cells of the M5 stimulation group were significantly increased compared with the normal group (P < 0.05), indicating successful activation of this signaling axis by inflammatory cytokines; the expression of these molecules was significantly decreased after OMT intervention (P < 0.05), returning to levels close to those of the normal group and the MTX positive control group. Meanwhile, the mRNA expression of the pathway negative feedback factor SOCS1 and the pro-apoptotic factor Bax was downregulated, while the mRNA expression of the anti-apoptotic factor Bcl-2 was upregulated in the M5 group; OMT intervention could reverse these abnormalities, making the expression of each molecule approach normal levels. Western blotting was used to detect the phosphorylation of pathway proteins and the expression of apoptosis-related proteins (Figures 9B,C). Compared with the M5 group, the protein expressions of p-JAK1, p-JAK2, p-STAT3 and EGFR were significantly decreased in the OMT group, MTX group and normal group (P < 0.05), while the protein expressions of SOCS1, Bax and CASP3 were significantly increased (P < 0.05). These results indicated that M5 stimulation could induce excessive activation of the EGFR-JAK/STAT pathway and inhibit apoptosis in HaCaT cells; OMT could effectively reverse these abnormalities, inhibit the activation of the signaling axis at the protein phosphorylation level, enhance SOCS1-mediated negative feedback regulation, and restore apoptotic execution function. These *in vitro* results were consistent with the trend of clinical skin lesion detection, further supporting the molecular mechanism that OMT restores the apoptotic homeostasis of keratinocytes by inhibiting the EGFR-JAK/STAT pathway.

**FIGURE 9:**
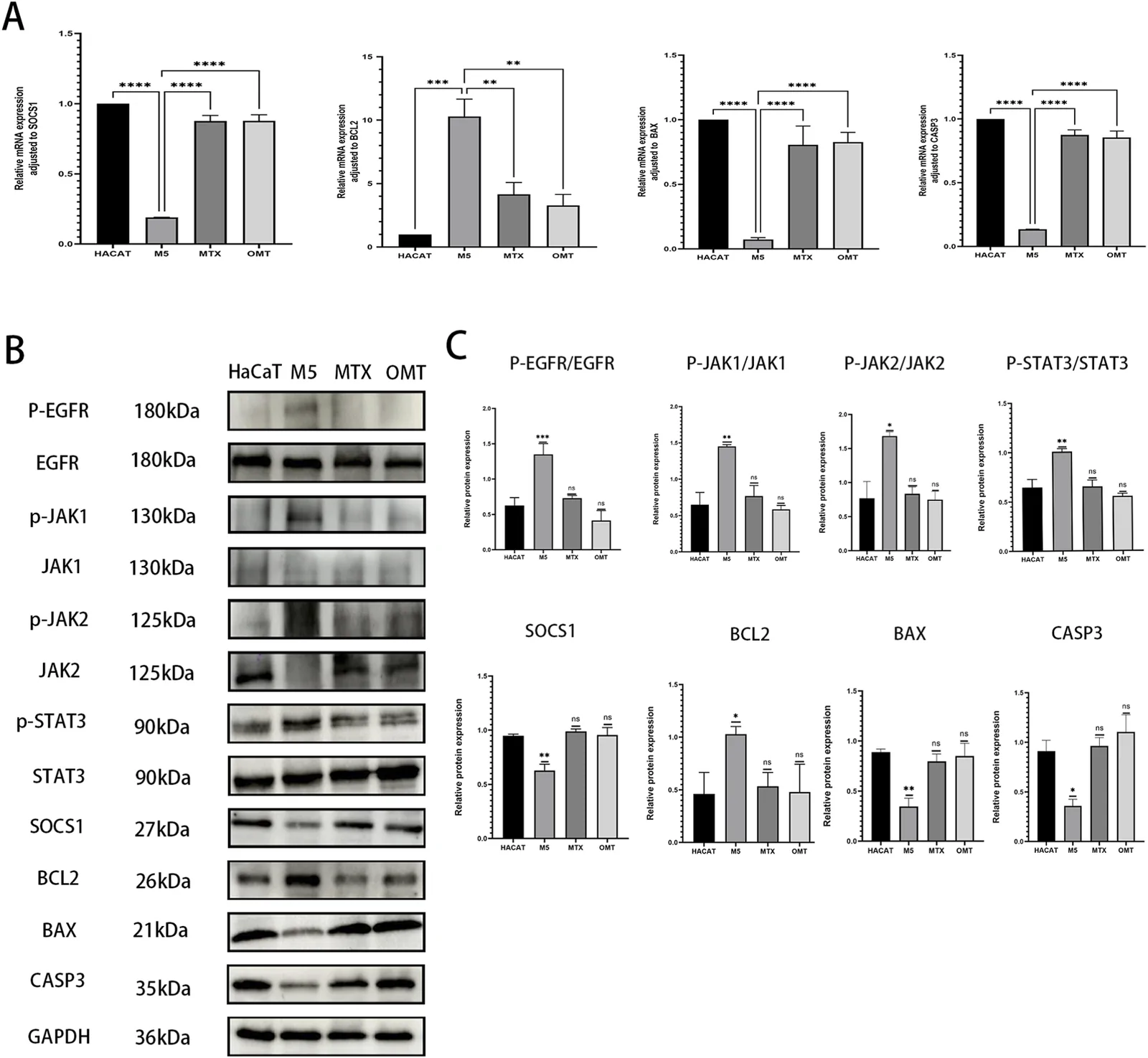
Regulatory effects of OMT on EGFR-JAK/STAT signaling pathway and related target protein expression in M5-induced psoriasis HaCaT cells. **(A)** Relative mRNA expression levels of SOCS1, BCL2, BAX and CASP3 detected by quantitative real-time polymerase chain reaction (qRT-PCR). **(B)** Expression of phosphorylated and total EGFR, JAK1, JAK2, STAT3, as well as SOCS1 and apoptosis-related proteins (BCL2, BAX, CASP3) detected by Western blotting. GAPDH was used as the internal reference. **(C)** Quantitative analysis of gray values of Western blot bands. n = 3, *P < 0.05, **P < 0.01, ***P < 0.001, ****P < 0.0001; ns: no statistically significant difference.

## 4 Discussion

Psoriasis is a chronic autoimmune skin disease involving multiple targets and signaling pathways. Although conventional immunosuppressants and biologics can alleviate clinical symptoms, adverse drug reactions and disease relapse after drug withdrawal greatly impair patient prognosis (Wang and Wang, 2025). OMT, an alkaloid extracted from the roots of *S. alopecuroides*, exerts pharmacological activities including anti-inflammatory and immunomodulatory effects. It has been clinically applied for the treatment of chronic hepatitis, yet the specific molecular mechanisms underlying its therapeutic effects on psoriasis remain poorly elucidated. Based on systems biology and bioinformatics, network pharmacology enables the characterization of the multi-component and multi-target properties of traditional Chinese medicine by analyzing the interactive networks among drugs, targets and diseases (Zhai et al., 2025).

Based on this technical approach, the present study screened potential therapeutic targets and systematically investigated the mechanism of action of OMT in the treatment of psoriasis by combining clinical skin lesion specimens and *in vitro* cell experiments, focusing on the EGFR-JAK/STAT signaling axis. By integrating multiple databases, we obtained 5,616 psoriasis-related targets and 241 OMT targets, and identified 109 common intersection targets. PPI network analysis confirmed that EGFR, AKT1, CASP3, MMP9 and BCL2 were core targets, among which EGFR and AKT1 occupied central positions in the regulatory network. Functional enrichment results showed that the core targets, with the JAK/STAT signaling axis as the hub, were involved in pathological processes such as signal transduction, apoptosis and inflammatory response. Combined with pathway enrichment, molecular docking and *in vivo* and *in vitro* validation experiments, it was confirmed that the EGFR-JAK/STAT signaling axis is the core pathway through which OMT exerts therapeutic effects on psoriasis. This pathway forms a complete regulatory hierarchy including receptor activation, kinase transduction, transcriptional regulation and negative feedback balance. Apoptosis-related proteins such as BCL2, Bax and CASP3 are regulated by this pathway, jointly mediating the disorder of keratinocyte apoptosis and participating in the pathogenesis of psoriasis.

As a key initiating factor of the pathway, EGFR is abnormally highly expressed in psoriasis skin lesion tissues. Inflammatory cells in skin lesions secrete cytokines such as IL-17A and TNF-α, which, together with EGF and TGF-α ligands, promote the dimerization and activation of EGFR (Lee et al., 2023), thereby phosphorylating the tyrosine sites of downstream JAK1 and JAK2 and initiating a signaling cascade. Meanwhile, activated EGFR can synergize with inflammatory cytokines to activate the PI3K/AKT pathway, forming a cross-talk loop with the JAK/STAT pathway, which jointly induces abnormal proliferation and inhibits apoptosis of keratinocytes. Molecular docking results showed that OMT could bind to the tyrosine kinase domain of EGFR with a binding energy as low as −8.3 kcal/mol, which could directly block protein activity and inhibit downstream kinase phosphorylation (Uribe and Gonzalez, 2011). Both clinical samples and cell experiments confirmed that the transcription, protein and phosphorylation levels of EGFR were synchronously downregulated after drug intervention, verifying that EGFR is a key upstream target of OMT.

JAK1 and JAK2 are core kinases of the pathway, which function cooperatively to mediate different inflammatory signals. JAK1 mainly transduces IFN-γ and IL-6 signals, while JAK2 is responsible for IL-17 and IL-23 signal transmission, jointly activating STAT3 and amplifying inflammatory and proliferative effects (Xue C. et al., 2023). The abnormal activation of the pathway is driven by upstream EGFR on the one hand, and on the other hand, it results from insufficient negative feedback regulation of SOCS1. Under normal physiological conditions, SOCS1 can bind to the JAK kinase domain to inhibit its activity. In psoriasis, the downregulation of SOCS1 expression fails to exert an inhibitory effect, leading to persistent activation of the pathway (Morelli et al., 2024). Our experiments found that the expressions of p-JAK1 and p-JAK2 were significantly increased in psoriasis skin lesions and inflammation-induced HaCaT cells, and were negatively correlated with SOCS1 expression. The binding energies of OMT with these two kinases were −7.7 kcal/mol and −8.0 kcal/mol, respectively. OMT could stably bind to the JAK catalytic domain and dually inhibit kinase activity, blocking signal transduction downstream, which has unique advantages over single-target inhibitors.

After phosphorylation modification, STAT3 undergoes nuclear translocation, regulates the expression of inflammation and proliferation-related genes, exacerbates local inflammatory responses, and forms a vicious cycle of inflammation and proliferation. Clinical detection showed that the expression of p-STAT3 was significantly increased in psoriasis skin lesions, and its expression level was correlated with the severity of skin lesions. OMT intervention could significantly reduce the phosphorylation level of STAT3, inhibit its nuclear transcription function, reduce the release of inflammatory factors, and alleviate skin inflammatory infiltration.

BCL2, Bax and CASP3 are key proteins regulating keratinocyte apoptosis and are closely related to the JAK/STAT pathway. In psoriasis tissues, BCL2 is abnormally highly expressed, while the expressions of Bax and CASP3 are decreased, resulting in blocked cell apoptosis. OMT could downregulate anti-apoptotic proteins and upregulate pro-apoptotic proteins, repair mitochondrial function, and promote the apoptosis of pathological keratinocytes. Molecular docking confirmed that the drug has binding activity with all three types of apoptotic proteins, which can directly regulate protein expression and synergistically correct the imbalance of cell apoptosis with the JAK/STAT pathway.

SOCS1 is an endogenous negative feedback regulator of the pathway, forming a closed-loop regulation with the JAK/STAT pathway. Moderate activation of the pathway can induce SOCS1 expression, which can bind to phosphorylated JAK proteins, inhibit kinase activity and promote their degradation (Tamiya et al., 2011). In psoriasis, activated STAT3 inhibits SOCS1 transcription, leading to its continuous downregulation and further exacerbating the abnormal activation of the pathway (Qin et al., 2017). OMT could significantly increase the expression level of SOCS1, which not only relieves the transcriptional inhibition of STAT3, but also indirectly regulates the NF-κB pathway to promote SOCS1 synthesis. The upregulated SOCS1 re-exerts its negative feedback function and inhibits JAK kinase activation, forming a positive regulatory loop, which is also an important molecular mechanism by which the drug reduces disease recurrence rate.

KEGG pathway enrichment suggested that OMT targets were also enriched in pathways such as apoptosis, necroptosis, rheumatoid arthritis and lipid and atherosclerosis. These pathways are interconnected through key molecules and form a complex regulatory network with the EGFR-JAK/STAT pathway, which is consistent with the multi-pathway pathogenesis characteristics of psoriasis. The apoptosis pathway has a bidirectional interaction with the core signaling axis: excessive activation of the JAK/STAT pathway disrupts the balance of apoptotic protein expression and inhibits cell apoptosis; activated CASP3 can in turn cleave JAK and STAT proteins, weakening pathway activity (Shen et al., 2023). OMT could bidirectionally regulate these two pathways, improve apoptotic disorders, and break the pathological vicious cycle.

The necroptosis pathway is mediated by inflammatory factors and interacts with the JAK/STAT pathway through JAK2 and BCL2. TNF-α and IFN-γ can simultaneously activate necroptosis and the JAK/STAT pathway, amplifying inflammatory damage (Woznicki et al., 2021); BCL2 can inhibit the necroptosis process, and STAT3 can upregulate BCL2 expression, hindering the clearance of pathological cells (Shi and Kehrl, 2019). OMT targets and regulates JAK kinases and BCL2 protein, inhibits excessive activation of the JAK/STAT pathway, downregulates BCL2 expression, relieves its inhibition on necroptosis, clears abnormally proliferating cells, and controls skin inflammatory damage.

The lipid and atherosclerosis pathway corresponds to the metabolic inflammatory lesions of psoriasis. Molecules such as oxLDL and ARL11 can cross-regulate the activity of the JAK/STAT pathway, and abnormal lipid metabolism will aggravate skin inflammation and cell proliferation (Shen et al., 2023). OMT acts on the JAK2 target to simultaneously improve skin lesions and lipid metabolism disorders, which also corroborates our previous findings on the drug’s role in regulating body metabolism (Xue et al., 2022).

In summary, the present study combined network prediction and *in vivo* and *in vitro* experiments to elucidate the core mechanism of OMT in the treatment of psoriasis. Targeting the EGFR-JAK/STAT signaling axis, the drug inhibits abnormal pathway activation and reduces local inflammatory responses from three levels: inhibiting receptor activation at the source, blocking kinase transduction in the middle, and upregulating negative feedback factors. Meanwhile, it directly regulates apoptosis-related proteins to restore the apoptotic homeostasis of keratinocytes. In addition, the drug links multiple cross-talk pathways such as apoptosis, necroptosis and lipid metabolism, and relies on the synergistic effects between molecules to comprehensively improve pathological damage in psoriasis and restore skin physiological homeostasis. This study clarifies the synergistic regulatory relationship between pathways and target proteins, fills the gaps in current research knowledge, improves the mechanistic theory of natural alkaloids in the intervention of autoimmune skin diseases, provides a new direction for targeted therapy of psoriasis, and offers theoretical support for the clinical application and new drug development of OMT. Future studies will construct animal models to explore the *in vivo* regulatory mechanism of the drug, screen differential target pathways using multi-omics technology, and carry out drug structure optimization and modification to improve targeting efficiency, providing ideas for the development of novel drugs.

## Data Availability

The raw data supporting the conclusions of this article will be made available by the authors, without undue reservation.
